# Different exercise training modalities produce similar endothelial function improvements in individuals with prehypertension or hypertension: a randomized clinical trial Exercise, endothelium and blood pressure

**DOI:** 10.1038/s41598-020-64365-x

**Published:** 2020-05-06

**Authors:** Marinei L. Pedralli, Rafael A. Marschner, Daniel P. Kollet, Salvador G. Neto, Bruna Eibel, Hirofumi Tanaka, Alexandre M. Lehnen

**Affiliations:** 1 Institute of Cardiology of Rio Grande do Sul/University Foundation of Cardiology, Porto Alegre, Brazil; 2 Thyroid Section, Endocrine Division, Hospital de Clínicas de Porto Alegre, Federal University of Rio Grande do Sul, Porto Alegre, Brazil; 3 Cardiovascular Aging Research Laboratory, Department of Kinesiology & Health Education, The University of Texas at Austin, Austin, TX USA

**Keywords:** Hypertension, Risk factors

## Abstract

Endothelial dysfunction is a characteristic of systemic arterial hypertension (SAH) and an early marker of atherosclerosis. Aerobic exercise training (AT) improves endothelial function. However, the effects of resistance training (RT) and combined training (CT) on endothelial function remain controversial in individuals with SAH. We determined the effects of AT, RT, and CT on endothelial function and systolic (SBP)/diastolic blood pressure (DBP) in individuals with prehypertension or hypertension. Forty-two participants (54 ± 11 y, resting SBP/DBP 137 ± 9/86 ± 6 mmHg) were randomly allocated into AT (n = 14, 40 min of cycling, 50–75% heart rate reserve), RT (n = 14, 6 resistance exercises, 4 × 12 repetitions, 60% maximum strength) and CT (n = 14, 2 × 12 repetitions of RT + 20 min of AT). All participants performed a 40-minute exercise session twice a week for 8 weeks. Endothelial function was evaluated by brachial artery flow-mediated dilation (FMD). Blood pressure was evaluated through ambulatory monitoring for 24 hours. After 8 weeks of exercise training, blood pressure was reduced in all 3 groups: −5.1 mmHg in SBP (95%CI –10.1, 0.0; p = 0.003) in AT; −4.0 mmHg in SBP (95%CI −7.8, −0.5; p = 0.027) in RT; and −3.2 mmHg in DBP (95%CI −7.9, 1.5; p = 0.001) in CT. All 3 exercise training modalities produced similar improvements in FMD: + 3.2% (95%CI 1.7, 4.6) (p < 0.001) in AT; + 4.0% (95%CI 2.1, 5.7) (p < 0.001) in RT; and +6.8% (95%CI 2.6, 11.1) (p = 0.006) in CT. In conclusion, different exercise training modalities were similarly effective in improving endothelial function but impacts on ambulatory blood pressure appear to be variable in individuals with prehypertension or hypertension.

## Introduction

Systemic arterial hypertension (SAH) is a highly prevalent cardiovascular risk factor^1^ and is associated with endothelial dysfunction^2,3^. Endothelial dysfunction is a phenotypic alteration in the endothelium characterized by prothrombotic, pro-inflammatory, and pro-constrictor conditions^4^. A reduction of 0.62% in endothelial function quantified by flow-mediated dilation (FMD) is associated with an increase of 20 mmHg in systolic blood pressure (SBP)^2^. Thus, given the relationship between SAH and endothelial dysfunction and high cardiovascular risk associated with SAH^5^, improving endothelial function and decreasing blood pressure (BP) are crucial for management and prevention strategies in individuals with prehypertension and hypertension^5^.

Regular aerobic exercise is a first-line strategy for preventing and treating essential hypertension^6,7^. Moderate-intensity aerobic exercise training increases nitric oxide (NO) bioavailability^8^, resulting in an improvement in endothelial function in normotensive individuals. Furthermore, improvements in endothelium-dependent vasodilation with regular aerobic exercise training (AT) are associated with lower blood pressure levels in hypertensive individuals^9,10^.

In a previous meta-analysis conducted by our group, we found 1 randomized controlled trial (RCT) that evaluated the effects of resistance training (RT) on endothelial function and 1 RCT with combined training (CT) on the same outcome^11^. Given the lack of RCTs on the effects of different exercise modalities on endothelial function, the clinical importance of endothelial function leading to cardiovascular risks, and conflicting results of the effectiveness of RT and CT on BP, we conducted a randomized clinical trial to assess the effects of AT, RT and CT on endothelial function and BP among adults with prehypertension or hypertension. Our primary outcome was endothelial function as measured by FMD, and secondary outcomes included ambulatory blood pressure, cardiac structure and function, maximal oxygen consumption, and maximum muscular strength. Our hypothesis was that RT and CT would elicit improvements in endothelial function that are comparable to AT.

## Methods

### Experimental protocol

The initial protocol of the present study entitled the Study of Endothelial Function Response to Exercise Training in Hypertensive Individuals (SEFRET) has been previously published^12^. SEFRET is a randomized, evaluator-blinded, parallel-group clinical trial with three different modes of exercise training used as interventions. The primary outcome measure was changes in endothelial function in response to different interventions. The secondary outcome was alterations in ambulatory BP monitoring (ABPM) for 24 hours. The project was approved by the Research Ethics Committee of the Institute of Cardiology of RS/University Foundation of Cardiology (ICFUC, Porto Alegre, RS, Brazil) and registered in the Brazilian Registry of Clinical Trials (Date: 01/03/2015; ID “RBR-9ygmdn”; http://www.ensaiosclinicos.gov.br/). The study follows the principles of the Declaration of Helsinki. All participants read and signed an informed consent form before participating in the study. Additionally, this study follows the recommendations proposed by the CONSORT *Statement*.

### Participants

The study participants were adults diagnosed with prehypertension or hypertension (resting SBP ≥ 130 mmHg or DBP ≥ 80 mmHg) according to the VI Brazilian Hypertension Society Guidelines^13^. The study was conducted from 4 May 2015 to February 2018 at a referral hospital in southern Brazil. Recruitment was through on-site screening, media postings, and patient databases. Forty-two volunteers with no history of cardiovascular (except for hypertension controlled with medication), neuromuscular, endocrine, and/or metabolic diseases, insufficiently active (<150 min/week or <600 MET-min/week) assessed by the International Physical Activity Questionnaire (IPAQ) (http://www.ipaq.ki.se/), physically capable of participating in an exercise program were eligible. To confirm the conditions of prehypertension or hypertension, all patients attending a first appointment at the hospital clinic had their blood pressure checked in the office. Those who met the inclusion criteria were referred to a cardiologist (DK or SGN) for further evaluation. Then an initial ABPM was performed and analyzed^15^. The diagnosis of prehypertension or hypertension was established through these 3 steps, and each patient had to go through three visits to confirm their entry in the study.

Blood sample was collected for fasting blood glucose concentrations, lipids and lipoproteins, and inflammatory markers. Exclusion criteria included lipid-lowering medications or estrogen replacement drugs; participation in any structured physical exercise program in the previous six months; type 1 or type 2 diabetes mellitus; smoking; grade II or III obesity (as measured by BMI), infectious diseases, chronic renal failure, or other malignant disease with life expectancy <2 years, or any other medical conditions that contraindicated for physical training.

The participants included in the study were assigned to one of the three groups: aerobic exercise training (AT, n = 14; 9 females and 5 males); resistance training (RT, n = 14; 6 females and 8 males); combined aerobic and resistance training (CT, n = 14; 8 females and 6 males). Randomization was performed through the website “www.randomization.org” with a coded distribution of a 1:1 ratio, with 3 blocks of 14 allocations. The participants were blinded to group allocations during the initial tests. The investigators responsible for the evaluations throughout the study were also blinded to group allocations.

The initial sample size^12^ included 81 individuals with prehypertension or hypertension (27 participants per group for 3 groups). After we obtained the preliminary results in FMD, we chose to recalculate the sample size for a 5% significance level, 80% power, and an expected difference of 2.5% (absolute FMD value). We arrived at the final sample size of 44 individuals with prehypertension or hypertension (~14 participants per group for 3 groups).

### Flow-mediated dilation

Endothelium-dependent brachial vascular function was evaluated using FMD according to established guidelines^14^. Briefly, the participants were asked to rest for 20 minutes in supine position and then a blood pressure cuff was placed on the proximal third of the arm (5 cm from the cubital fossa). Baseline longitudinal brachial artery diameters were measured along with pulsed Doppler signals for flow velocity analysis. After baseline recordings were completed, reactive hyperemia was induced by inflation of a blood pressure cuff to 50 mmHg above previously-measured SBP, and cuff inflation was maintained for 5 minutes. Two-dimensional images of the brachial artery were acquired using a linear-array multi-frequency transducer (7–12 MHz) connected to a high-resolution ultrasound system (EnVisor Series, Philips Medical System; Bothell, WA, USA) and ECG recording system. The time of each image acquisition during the cardiac cycle was determined from simultaneous ECG recording. During image acquisition, anatomical landmarks such as veins and fascial planes were observed to help maintain the same image of the artery throughout the study. The longitudinal image of the brachial artery was recorded continuously for 30 seconds before (baseline image) to 2 minutes after cuff deflation (peak diameter). FMD was expressed as the percentage change in arterial diameter from baseline [FMD (%) = ((peak diameter − baseline diameter) / baseline diameter) × 100]. Blood flow velocity was assessed at baseline and post-reactive hyperemia. Then the percentage change in post-hyperemia blood flow velocity from baseline was calculated [blood flow velocity (%) = (reactive hyperemia blood flow velocity − resting blood flow velocity)/resting blood flow velocity × 100]. All analyses were performed offline by a blinded evaluator. All participants were studied in the morning after an overnight fast and they were asked to avoid vasoactive substances (drugs, caffeine and alcohol). Given that vascular reactivity may vary throughout the normal menstrual cycle, female participants were studied during the luteal phase^15^.

### Ambulatory blood pressure

Blood pressure monitoring devices (Spacelab, Redmond, WA, USA) were used to obtain 24-hour BP recordings following standard procedures^16^. During ABPM, BP was recorded every 15 minutes between 7:00 a.m. and 10:00 p.m. and every 30 minutes during night time (from 11:00 p.m. to 6:00 a.m.). We considered an average sleep time of eight hours based on the participant’s reported daily routine in the initial interview. The final ambulatory BP values were considered valid if 85% of all readings were available. When there were no ABPM readings for a full hour, the average values for the hour immediately before and after were recorded. The participants were asked to perform their daily activities as usual. They were asked to keep a detailed record of their daily activities such as sleep, work, leisure, food, among others, for verification of any sudden changes in BP. ABPM measures were assessed by an evaluator blinded to the study interventions.

### Echocardiography

Participants were examined at rest by an experienced cardiologist using two-dimensional echocardiography (Phillips IEE 33). Left ventricular volume (LV) and ejection fraction (EF) were calculated from the apical four- and two-chamber views, using the modified Simpson biplane method. All measurements of echocardiography were evaluated by a blinded evaluator.

### Functional capacity and peak muscle strength

Participants performed maximal exercise tests on a treadmill following the Bruce protocol^17^. The test was performed under the direct supervision by a cardiologist (blinded for all participants). Maximum oxygen consumption (VO_2_max) was predicted based on total exercise time according to the Bruce protocol and presented in the final report. The test results were used to set the initial intensity of the exercise training for the AT and CT groups. Peak muscle strength was determined using the one-repetition maximum (1-RM) test^18^ on the testing equipment with guided loads (Moviment®) for the following exercises: leg press, bench press, extension of the knee, direct biceps threading, knee flexion and low row. All 1-RM tests were conducted by Physical Education professionals in the presence of a cardiologist within the ICFUC Clinical Research Laboratory. The details of the protocol were published previously^12^. The 1-RM test results were used to set the initial training intensity for the RT and CT groups.

### Exercise training

The aerobic exercise training group performed 40 minutes of aerobic exercise on a cycle ergometer, with progressive intensity of 50–75% heart rate reserve or 11–14 of the rating of perceived exertion scale^19^ for volunteers using β-blockers (Table 1). Strength training consisted of 4 sets of 8–12 repetitions, with 60–80% 1-RM intensity for leg press, bench press, knee extension, biceps direct threading, knee flexion and low row (total time: ~40 minutes). The combined training consisted of RT (same exercises but with 2 sets/12 repetitions, totaling 20 minutes, with intensity between 60–80% of 1-RM) + AT (20-minute length, with intensity from 50–75% heart rate reserve). Some parameters of exercise training have been modified from the previously published procedures^12^. The total length, initially designed for 12 weeks, was adjusted to 8 weeks. The frequency of 3 times a week, was reduced to 2 times a week. All participants were instructed to maintain their usual lifestyles including eating habits.

**Table 1:** Characteristics of patients randomized to aerobic, resistance, and combined training.

	Aerobic Training (n = 13)			Resistance Training (n = 12)			Combined Training (n = 12)			p (group)	p (time)	p (interaction)
	Pre	Post	∆	Pre	Post	∆	Pre	Post	∆			
Age (year) (95% CI)	50.9 ± 14.2 (45.2, 56.7)		—	55.1 ± 6.9 (52.2, 58.0)		—	53.8 ± 9.1 (49.9, 57.7)		—			
Height (cm) (95% CI)	163 ± 8 (159, 166)		—	168 ± 10 (164, 172)		—	166 ± 12 (161, 172)		—			
Body mass (kg) (95% CI)	79.0 ± 12.9 (72.3, 85.8)	77.8 ± 12.9 (71.1, 84.6)	−1.2 (−2.0, −0.4)	81.4 ± 22.3 (68.9, 93.2)	81.0 ± 22.0 (69.0, 92.9)	−0.4 (−1.1, 1.0)	78.2 ± 23.3 (65.5, 90.8)	77.0 ± 23.9 (65.5, 90.8)	−1.2 (−1.8, −0.5)	0.915	0.002	0.171
BMI (kg/m^2^) (95% CI)	29.8 ± 4.1 (27.7, 32.0)	29.3 ± 4.2 (27.2, 31.6)	−0.5 (−0.8, −0.1)	28.5 ± 6.01 (25.2, 31.7)	28.4 ± 5.8 (25.3, 31.6)	−0.1 (−0.4, 0.4)	27.9 ± 5.5 (24.8, 30.9)	27.3 ± 5.8 (24.3, 30.6)	−0.6 (−0.7, −0.2)	0.573	0.001	0.115
Waist (cm) (95% CI)	92.4 ± 8.1 (88.2, 96.7)	88.3 ± 9.2a^†^ (83.6, 90.1)	−4.1 (−6.1, −2.0)	92.7 ± 16.5 (83.8, 101.7)	90.7 ± 15.1b (82.5, 98.9)	−2.0 (−6.1, 2.0)	89.6 ± 14.5 (81.9, 97.7)	86.5 ± 15.0a^†^ (78.9, 92.3)	−3.1 (−4.0, −1.5)	0.855	<0.001	0.043
VO_2_max (ml/kg/min) (95% CI)	31.1 ± 12.3 (24.7, 37.6)	38.2 ± 10.4^†^ (31.3, 45.2)	7.1 (4.1, 10.0)	35.5 ± 5.2 (32.4, 38.5)	39.2 ± 5.3^†^ (36.2, 42.0)	3.7 (0.6, 6.3)	37.5 ± 7.9 (33.0, 41.6)	38.2 ± 4.5 (36.0, 40.9)	0.7 (−3.0, 5.3)	0.629	<0.001	0.049
Ejection fraction (%) (95% CI)	61.6±11.2 (55.7, 67.5)	67.6±11.5a^†^ (61.6, 73.7)	6.0 (0.9, 11.1)	68.1±8.5 (62.8, 73.6)	61.2±19.8b (53.9, 71.4)	−6.9 (−16.5, 5.5)	67.5±9.18 (63.5, 72.4)	65.2±11.0b (45.8, 70.6)	−2.3 (−10.4, 2.8)	0.871	0.292	0.022
HbA1C (%) (95% CI)	5.7 ± 0.5 (5.5, 0.1)	5.6 ± 0.5 (5.4, 5.9)	−0.1 (−0.3, 0.1)	5.6 ± 0.3 (5.4, 5.8)	5.5 ± 0.3 (5.3, 5.7)	−0.1 (−0.8, 0.4)	5.8 ± 0.3 (5.6, 6.0)	5.6 ± 0.5 (5.4, 5.9)	−0.2 (−0.3, −0.1)	0.401	0.027	0.724
Creatinine (mmol/L) (95% CI)	0.065 ± 0.014 (0.059, 0.073)	0.068 ± 0.015 (0.060, 0.076)	0.003 (−0.002, 0.006)	0.077 ± 0.015 (0.068, 0.084)	0.080 ± 0.014 (0.070, 0.088)	0.003 (−0.002, 0.005)	0.082 ± 0.025 (0.070, 0.097)	0.079 ± 0.025 (0.069, 0.096)	−0.003 (−0.005, 0.002)	0.057	0.546	0.207
Total-C (mmol/L) (95% CI)	5.48 ± 1.09 (4.91, 6.03)	5.15 ± 0.98 (4.65, 5.66)	−0.33 (−0.70, 0.07)	4.97 ± 1.11 (4.27, 5.46)	5.12 ± 0.96 (4.60, 5.66)	0.15 (−0.12, 0.68)	4.60 ± 0.96 (4.24, 5.28)	4.91 ± 0.78 (4.50, 5.33)	0.31 (−0.36, 0.70)	0.334	0.736	0.089
HDL-C (mmol/L) (95% CI)	1.37 ± 0.28 (1.22, 1.50)	1.40 ± 0.31 (1.24, 1.55)	0.03 (−0.04, 0.11)	1.29 ± 0.39 (1.00, 1.45)	1.27 ± 0.41 (1.06, 1.53)	−0.02 (−0.03, 0.12)	1.29 ± 0.41 (1.11, 1.55)	1.37 ± 0.39 (1.14, 1.55)	0.08 (0.06, 0.09)	0.721	0.133	0.444
LDL-C (mmol/L) (95% CI)	3.18 ± 1.03 (2.66, 3.72)	3.03 ± 0.93 (2.53, 3.52)	−0.15 (−0.56, 0.25)	2.95 ± 0.03 (2.46, 3.28)	3.18 ± 0.72 (2.79, 3.57)	0.23 (0.01, 0.63)	2.69 ± 1.06 (2.12, 3.26)	2.82 ± 0.93 (2.33, 3.34)	0.13 (−0.33, 0.60)	0.578	0.401	0.194
TG (mmol/L) (95% CI)	4.60 ± 2.17 (3.46, 5.72)	3.57 ± 1.19^†^ (3.00, 4.14)	−1.03 (−1.97, −0.10)	3.67 ± 1.78 (2.82, 4.76)	3.31 ± 1.14^†^ (2.69, 3.93)	−0.36 (−1.06, 0.12)	3.67 ± 2.59 (2.15, 4.97)	3.26 ± 2.92^†^ (2.12, 5.30)	−0.41 (−0.51, 0.63)	0.604	0.029	0.047
**Antihypertensive drugs**												
β-blocker, n (%)	4 (30.8)		2 (16.6)			3 (25.0)						
ACE inhibitor, n (%)	6 (46.2)		1 (8.3)			2 (16.6)						
Antiplatelet, n (%)	2 (15.4)		1 (8.3)			—						
Diuretics, n (%)	8 (61.5)		7 (58.3)			5 (41.7)						
ARBs, n (%)	8 (61.5)		8 (66.6)			6 (50.0)						

Data are means ± SD. BMI: body mass index; VO2max: maximal oxygen consumption predicted by the Bruce protocol; HbA1C: glycated hemoglobin A1C; C: cholesterol; HDL: high-density lipoprotein; LDL: low-density lipoprotein; TG: Triglycerides; ACE: angiotensin-converting-enzyme; ARBs: angiotensin II receptor blockers. Some individuals were using more than one drug. The differences were tested using the Generalized Estimating Equations (GEE) with two factors (exercise modality and time) as well as the interaction between them. Multiple comparisons, when applicable, were tested by Bonferroni. The differences between groups in the same period of time are represented by letters. Thus, equal letters have no difference between values (p < 0.05). The differences between initial and final measurements in the same group are represented by ^†^ (p < 0.05).

### Statistical analyses

The normality of data was tested using Shapiro-Wilk tests. The differences among groups were tested using the Generalized Estimating Equations (GEE) with two factors (exercise modality and time) as well as the interaction between them. Multiple comparisons, when applicable, were tested by Bonferroni. Our study included female and male participants and our results were compared by gender using the chi-square test. Cohen’s effect size was also applied to FMD measurements (pre- *versus* post-exercise training) and classified as small (0.20 to 0.49), medium (0.50 and 0.79), and large (above 0.80)^20^. For the ambulatory BP values, two analyses were performed. First, mean values of SBP and DBP for daytime period (7–22h), nighttime (23–6h), and the total of 24 h, both at baseline and at the end of the 8-week exercise training were computed. The times were chosen based on comparability with the literature^21^. A second analysis was performed considering the differences (Δ) between the initial and final values for each hour of the monitoring period. Values were presented as means ± SD and/or 95% confidence interval (95%CI). Statistical analyses were performed using the SPSS software (version 23.0, Chicago, Illinois) with α ≤ 0.05.

## Results

**Figure 1:**
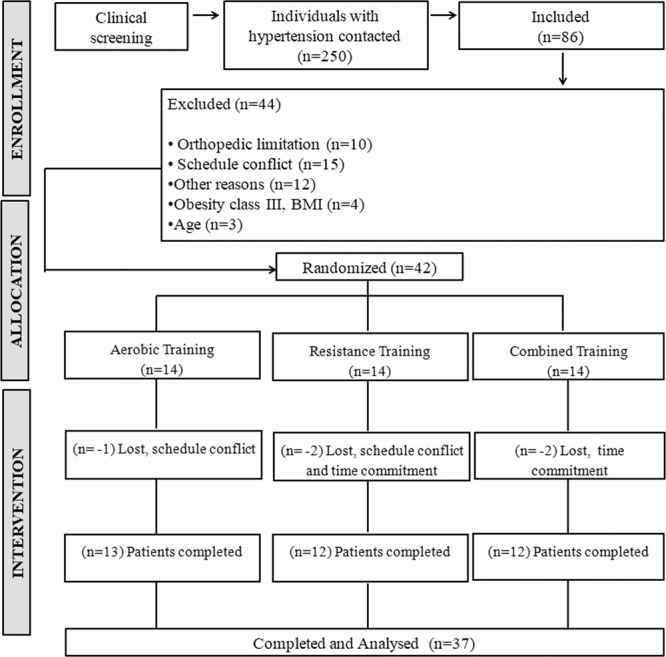
Flow diagram of the SEFRET study.

Following the initial screening, 86 participants met the inclusion criteria and were invited to an interview for inclusion/exclusion criteria verifications. Forty-four participants were found ineligible to participate. Of the 42 eligible participants, five did not complete the training protocol. Therefore, a total of 37 participants comprising 21 (56.8%) females and 16 (43.2%) males were included in the analyses (Fig. 1). There was a similar proportion of female and male participants (p = 0.163). Resting systolic and diastolic BP values (obtained by the study cardiologist) were 137 ± 10 (95%CI 133, 140) and 87 ± 12 mmHg (95%CI 84, 91), respectively.

The selected characteristics of the participants are shown in Table 1. No significant baseline differences were observed among groups for these characteristics. All participants reported low levels of physical activity: 570 MET-min/week. In addition, all drugs used by the participants are shown in Table 1. After an 8-week exercise training, no change was observed in body mass. However, significant reductions in waist circumference (p < 0.001) were noted for the AT and CT groups (Table 1). VO_2_max predicted by the Bruce protocol increased in the AT and RT groups, but not in the CT group. Ejection fraction increased in AT group (p = 0.038) whereas ejection fraction tended to decrease in the RT group. Serum triglyceride concentrations decreased in all 3 groups (p = 0.047). As shown in Tables 2, 1-RM muscle strength increased in all resistance exercises in the RT group, except in low rows that all groups increased similarly.

**Table 2:** Changes in one repetition maximum (1-RM) muscle strength in response to aerobic, resistance, and combined training.

	Aerobic Training (n = 13)			Resistance Training (n = 12)			Combined Training (n = 12)			p (group)	p (time)	p (interaction)
	Pre	Post	∆	Pre	Post	∆	Pre	Post	∆			
Leg press (kg)(95% CI)	67.4±17.3(58.3, 76.4)	80.7±17.9b^†^(71.3, 90.1)	13.3(10.2, 16.5)	74.5±27.5(59.6, 89.5)	99.7±30.7a^†^(83.1, 116.5)	25.2(19.2, 31.4)	64.2±13.7(56.8, 71.6)	87.2±15b^†^(79.0, 95.3)	23.0(19.7, 26.2)	0.345	<0.001	<0.001
Knee flexion (kg)(95% CI)	15.8±6.7(12.4, 19.1)	19.7±7.9b(15.5, 23.8)	3.9(2.4, 5.4)	16.6±6.4(13.1, 20.1)	25.3±8.8a^†^(20.5, 30.1)	8.7(5.9, 11.4)	16.8±6.1(13.4, 20.1)	22.1±5.8b(18.9, 25.2)	5.3(4.4, 6.2)	0.512	<0.001	0.010
Knee extension (kg) (95% CI)	29.7±10.6(24.2, 35.2)	34.8±12.9b^†^(28.1, 41.5)	5.1(3.4, 6.8)	42.1±17.2(32.8, 51.5)	53.7±19.3a^†^(43.3, 64.3)	11.6(7.3, 16.1)	35.7±12.7(28.8, 42.7)	49.7±22.5a^†^(37.5, 61.9)	14.0(8.1, 19.8)	0.015	<0.001	0.001
Bench press (kg)(95% CI)	17.4±8.0(13.2, 21.6)	21.9±9.8b(16.8, 27.1)	4.5(3.1, 5.9)	20.4±11.8(14.1, 26.9)	30.1±17.0a^†^(20.9, 39.4)	9.7(5.6, 13.7)	15.9±7.2(12.0, 19.8)	24.4±9.9b^†^(19.1, 29.8)	8.5(6.3, 10.8)	0.447	<0.001	0.002
Biceps curl (kg)(95% CI)	26.3±10.2(21.0, 31.6)	31.4±11.9b(25.2, 37.7)	5.1(3.3, 7.0)	38.2±14.6(30.3, 46.1)	48.1±17.4a^†^(38.6, 57.5)	9.9(6.7, 12.9)	27.6±9.2(22.6, 32.6)	35.3±9.9b^†^(30.0, 40.7)	7.7(6.2, 9.3)	0.023	<0.001	0.021
Low rows (kg)(95% CI)	71.1±19.8(60.7, 81.5)	86.5±24.9^†^(73.5, 99.5)	15.4(10.5, 20.3)	79.5±27.0(64.9, 94.2)	99.1±29.9^†^(82.9, 115.4)	19.6(14.7, 24.5)	69.1±14.5(61.1, 76.9)	88.7±15.5^†^(80.4, 97.2)	19.6(17.0, 22.5)	0.464	<0.001	0.030

Data are means ± SD. The differences were tested using the Generalized Estimating Equations (GEE) with two factors (exercise modality and time) as well as the interaction between them. Multiple comparisons, when applicable, were tested by Bonferroni. The differences between groups in the same period of time are represented by letters. Thus, equal letters have no difference between values (p < 0.05). The differences between initial and final measurements in the same group are represented by ^†^(p < 0.05).

**Figure 2:**
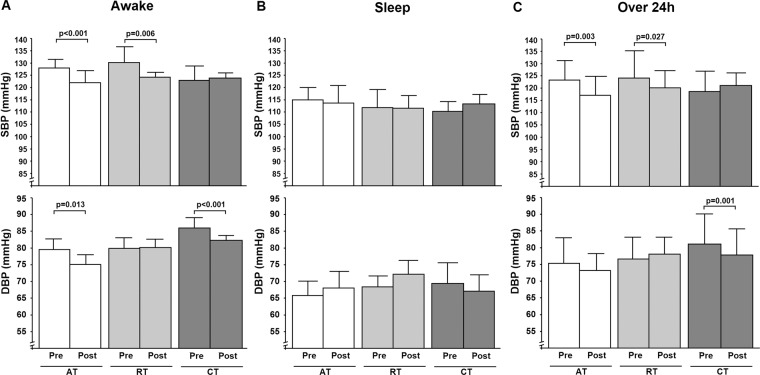
Ambulatory blood pressure in awake (Panel A), sleep (Panel B) and over 24-hour (Panel C) periods. Data are means ± SD. AT, aerobic training; RT, resistance training; CT, combined training.

**Figure 3:**
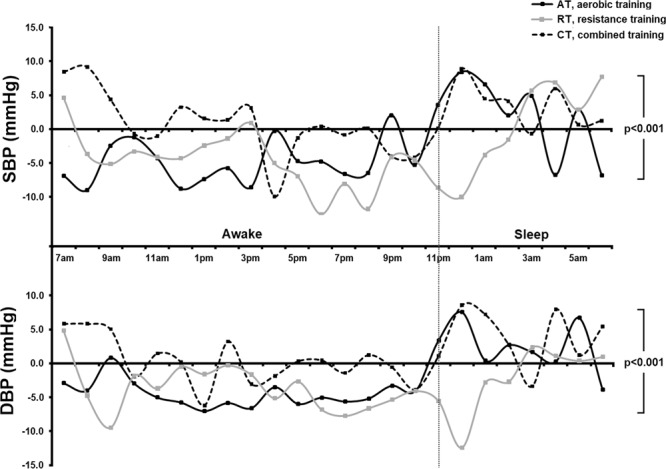
Change in blood pressure over 24-hour monitoring.

Figure 2 shows that over the 24-hour period, SBP decreased in the AT group by Δ5.1 mmHg (95%CI –10.1, 0.0; p = 0.003) and in the RT group by Δ4.0 mmHg 95%CI (–7.8, –0.5; p = 0.027), but not in the CT group. There was no significant difference in the magnitude of SBP reductions between the AT and RT groups. DBP decreased only in the CT group by Δ3.2 mmHg (95%CI −7.9, 1.5; p = 0.001). Awake or daytime SBP decreased in the AT and RT groups while DBP decreased in the AT and CT groups. During the night-time, no change was observed in blood pressure in any of the groups. Figure 3 shows the differences (Δ) in 24-hour blood pressure between the baseline and final values.

**Figure 4:**
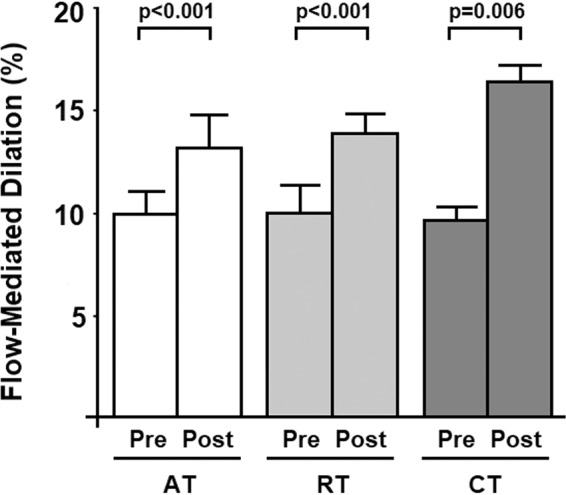
Flow-mediated dilation, an index of endothelium-dependent vasodilation, before (pre) and after (post) aerobic exercise training (AT), resistance training (RT), and combined training (CT). The results are expressed as mean ± SD.

The parameters of endothelium-dependent vasodilation are described in Table 3 and Fig. 4. At baseline, FMD was not significantly different between the groups. All the exercise training groups increased FMD significantly (Fig. 4). FMD increases were Δ3.2% (95%CI 1.7, 4.6; p < 0.001) for the AT group; Δ4.0% (95%CI 2.1, 5.7; p < 0.001) for the RT group; and Δ6.8% (95%CI 2.6, 11.1; p = 0.006) for the CT group. Cohen’s effect sizes were medium (0.6) for AT, large (0.9) for RT, and large (0.9) for CT. There were no significant group differences in the magnitude of increases in FMD (p = 0.248). Individual data points for FMD measurements are shown in Fig. S1 (supplementary material).

**Table 3:** Changes in arterial and hemodynamic measures in response to aerobic, resistance, and combined training.

	Aerobic Training (n=13)			Resistance Training (n=12)			Combined Training (n=12)			p (group)	p (time)	p (interaction)
	Pre	Post	∆	Pre	Post	∆	Pre	Post	∆			
**Brachial diameter**												
Resting (mm)	0.36 ± 0.06	0.38 ± 0.07	0.02	0.36 ± 0.07	0.37 ± 0.06	0.01	0.37 ± 0.08	0.39 ± 0.09	0.02	0.863	0.001	0.532
(95%CI)	(0.33, 0.39)	(0.34, 0.41)	(−0.00, 0.03)	(0.32, 0.40)	(0.33, 0.40)	(0.00, 0.02)	(0.32, 0.42)	(0.34, 0.44)	(0.00, 0.04)			
Peak (mm)	0.40 ± 0.06	0.43 ± 0.06	0.03	0.39 ± 0.07	0.41 ± 0.07	0.02	0.41 ± 0.09	0.45 ± 0.10	0.04	0.778	<0.001	0.448
(95%CI)	(0.37, 0.43)	(0.39, 0.45)	(0.00, 0.04)	(0.35, 0.43)	(0.38, 0.45)	(0.01, 0.03)	(0.36, 0.45)	(0.39, 0.50)	(0.01, 0.07)			
**Blood flow (BF) velocity**												
Resting (cm/s)	13.6 ± 7.0	21.0 ± 10.7	7.4	14.7 ± 7.8	19.5 ± 8.6	4.8	14.5 ± 9.9	23.7 ± 18.3	9.2	0.898	<0.001	0.686
(95%CI)	(9.9, 17.2)	(15.6, 27.5)	(1.7, 14.2)	(10.5, 19.0)	(14.9, 24.2)	(1.2, 8.4)	(10.5, 21.5)	(12.6, 31.4)	(−2.8, 14.9)			
Peak (cm/s)	100.0 ± 44.	104.4 ± 22.8	4.4	78.1 ± 26.2	98.6 ± 38.6	20.5	96.6 ± 28.2	113.1 ± 32.1	16.5	0.291	0.068	0.239
(95%CI)	0(75.5, 124.6)	(91.3, 110.3)	(−27.9, 19.5)	(63.9, 92.4)	(77.1, 119.6)	(3.1, 37.9)	(81.3, 111.9)	(95.6, 130.4)	(−2.8, 35.8)			
∆BF velocity (%)	887 ± 216	515 ± 133	−372	596 ± 115	499 ± 108	−97	815 ± 186	623 ± 105	−170	0.507	0.016	0.451
(95%CI)	(462, 1307)	(248, 769)	(−781, 28)	(371, 821)	(286, 711)	(−276, 81)	(450, 1180)	(421, 831)	(−501, 122)			

Data are means  ±  SD. The differences were tested using the Generalized Estimating Equations (GEE) with two factors (exercise modality and time) as well as the interaction between them. Multiple comparisons, when applicable, were tested by Bonferroni (p < 0.05).

## Discussion

The main finding of the present study is that different modalities of exercise training produced similar and consistent improvement in endothelium-dependent vasodilation as measured by flow-mediated dilation. These findings are consistent with our hypothesis that RT and CT would elicit improvements in endothelial function that are comparable to AT. Importantly, the improvements in vascular function were achieved with very mild amount of exercise (twice a week) for a short time period (8 weeks). Additionally, 24-hour SBP decreased with AT and RT, but not with CT. To the best of our knowledge, only two RCTs examined the chronic effects of different exercise modalities on endothelial function in individuals with hypertension. Our present results in middle-aged and older individuals with prehypertension or hypertension bring new clinical insight to combat endothelial dysfunction as an important cardiovascular risk factors.

FMD has an independent predictive capacity for future cardiovascular events^22,23^. In healthy and asymptomatic adults, a 1% increase in FMD is associated with a 4% reduction in cardiovascular risk^24^. In individuals with increased cardiovascular risk, such as hypertensives, the risk reduction may be greater as a 1% increase in FMD is associated with a 13% reduction in cardiovascular risk^22^. In the present study, improvements in FMD were 3.2% for aerobic training with Cohen’s effect size of 0.6, 4.0% for resistance training with Cohen’s effect size of 0.9, and 6.8% for combined training with Cohen’s effect size of 0.9. These magnitude of improvements in endothelial function should translate to clinically significant reductions in cardiovascular risks. Interestingly, combined training showed the greatest increase in FMD even though it did not produce a hypotensive effect on SBP.

To date, only two studies have investigated the effects of dynamic resistance training on endothelial function in individuals with high blood pressure. A previous study of resistance training performed for 60 min/session, three times a week for 8 weeks observed a small increase in FMD (2.1%) with a reduction in both SBP and DBP by 10/8 mmHg^25^. Our present study demonstrated a better benefit (4.0%) even with a lower amount of daily exercise training. Additionally, our combined training showed more marked benefits (6.8%) on endothelial function. In a meta-analysis on the effect of different modalities of exercise training on endothelial function^26^, combined aerobic and resistance training increased FMD by 2.1% in a total of 2,236 participants, including diabetics, transplant patients, hypertensive patients, chronic cardiac patients, pregnant women, postmenopausal women, and the metabolic syndrome. In the present study, we studied and analyzed only participants with prehypertension or hypertension^26^. It is plausible and likely that the magnitude of FMD improvement might be variable in different populations.

Physiological mechanisms by which regular exercise improves endothelial function are attributed to repeated perfusion leading to a higher shear stress in the vascular wall^27–29^. This mechanism is clearly applicable to the practice of aerobic exercises^30^. However, it is not clear what mechanism cold explain improvements in endothelial function induced by resistance training. Mechanical compression of the skeletal muscles involved in resistance exercise has been hypothesized to cause excessive vascular tension^31^, which could inflict an injury on the endothelium. However, an increase in reactive hyperemia has been reported in forearm after 6 months of strength training in young healthy adults^32^. During the execution of strength training, mechanical compression of resistance vessels induced by muscle contractions produce transient ischemia. Upon muscle relaxation, the release of blood flow produces hyperemia and the subsequent increase in shear stress^33^. Thus, although the stimuli may be different, both aerobic exercise and resistance training appear to produce similar benefits to the endothelium.

A variety of medical organizations recommend regular physical exercises as an initial lifestyle therapy for individuals with hypertension^34^. The results of this RCT showed reductions in blood pressure in individuals with prehypertension or hypertension after 8 weeks of twice-a-week moderate intensity exercises. This magnitude of blood pressure reductions can lower the risk of stroke by 14%^35^, coronary artery disease by 9%, and total mortality by 7%^34^. Furthermore, blood pressure reductions in response to physical training^34,36,37^ appear similar in magnitude to those obtained with first-line antihypertensive drugs^38^. Our ambulatory BP findings also suggest that the different types of exercises seem to positively influence BP values during the day and with no alteration at night.

Aerobic exercise training produced significant changes in body composition, maximum oxygen consumption, and lipid profile whereas resistance training resulted in increases in peak muscle strength. Enhancement and maintenance of muscle strength are the primary strategy for better performance of daily activities, reduced risk of falls, decreased musculoskeletal injuries, and incidence of osteoporosis for middle-aged and elderly individuals^39^. However, increases in muscle strength cannot be achieved with aerobic exercise training necessitating the performance of combined training that encompass both aerobic exercise and resistance training. A controversy regarding the effects of combined training on blood pressure appears to exist^9^. In the present study, combined training showed hypotensive effects only on DBP similarly to a previous clinical trial^9^. However, reductions in 5/4 mmHg in SBP/DBP have been reported in elderly hypertensive patients who completed a combined training protocol for 6 months^40^. It is possible that a longer duration of combined training might be necessary to induce significant reductions in SBP.

Some limitations of this study should be noted and emphasized. First, a time control group (without exercise training) was not included. The implementation of sedentary controls may be considered unethical because patients with hypertension who are at high risk of developing cardiovascular disease would not be treated. Since the intervention consists of chronic exercise training, we have decided that aerobic exercise training is the gold standard strategy (defined in the innumerable guidelines) and compare RT and CT to this first-line strategy. Second, the sample size in each group is relatively small. Our small sample size limits the analyses with acceptable sensitivity stratified by sex (gender) and/or medication use. However, even with a relatively small sample, we have demonstrated clinically important changes in FMD (primary outcome) by mean differences and Cohen’s effects.

In summary, we concluded that different moderate-intensity exercise modalities promoted significant improvement in endothelial function (AT 3.2%, RT 4.0% and CT 6.8%) in eight months of exercise training. Our results are clinically relevant because an increase of 1% in FMD is associated with a 13% reduction of cardiovascular risk in individuals with increased cardiovascular risk such as those suffering from hypertension^22^. Furthermore, the reduction in SBP levels in individuals with prehypertension or hypertension (AT 5.1 mmHg and RT 4.0 mmHg) is also a major finding that is within the expected range of BP reductions^7^.

## Supplementary information

Supplementary information.
