# Publisher Correction: Different exercise training modalities produce similar endothelial function improvements in individuals with prehypertension or hypertension: a randomized clinical trial

**DOI:** 10.1038/s41598-020-67586-2

**Published:** 2020-06-24

**Authors:** Marinei L. Pedralli, Rafael A. Marschner, Daniel P. Kollet, Salvador G. Neto, Bruna Eibel, Hirofumi Tanaka, Alexandre M. Lehnen

**Affiliations:** 1 Institute of Cardiology of Rio Grande do Sul/University Foundation of Cardiology, Porto Alegre, Brazil; 2 Thyroid Section, Endocrine Division, Hospital de Clínicas de Porto Alegre, Federal University of Rio Grande do Sul, Porto Alegre, Brazil; 3 Cardiovascular Aging Research Laboratory, Department of Kinesiology & Health Education, The University of Texas at Austin, Austin, TX USA

Correction to: *Scientific Reports* https://doi.org/10.1038/s41598-020-64365-x, published online 06 May 2020

The original version of this Article contained an error in the title, where “Exercise, endothelium and blood pressure” was inadvertently included at the end of the title.

Additionally, this Article contained an error in the Discussion section.

“In summary, we concluded that different moderate-intensity exercise modalities promoted significant improvement in endothelial function (AT 3.2%, RT 4.0% and CT 6.8%) in eight months of exercise training.”

now reads:

“In summary, we concluded that different moderate-intensity exercise modalities promoted significant improvement in endothelial function (AT 3.2%, RT 4.0% and CT 6.8%) in eight weeks of exercise training.”

These errors have now been corrected in the PDF and HTML versions of the Article.

