# Supplementary material for: Different exercise training modalities produce similar endothelial function improvements in individuals with prehypertension or hypertension: a randomized clinical trial Exercise, endothelium and blood pressure
Source: Sci Rep. 2020 May 6;10:7628. doi: 10.1038/s41598-020-64365-x (PMC7203179; doi:10.1038/s41598-020-64365-x)
Supplement: Supplementary file 1 — Supplementary information. [file 41598_2020_64365_MOESM1_ESM.pdf]

Supplementary Material

Different exercise training modalities produce similar endothelial function improvements in individuals with prehypertension or hypertension: a randomized clinical trial

Marinei L Pedralli[1], Rafael A Marschner[2], Daniel P Kollet[1], Salvador G Neto[1], Bruna Eibel[1], Hirofumi Tanaka[3], Alexandre M Lehnen[1*]

[1] Institute of Cardiology of Rio Grande do Sul/University Foundation of Cardiology, Porto Alegre, Brazil.

[2] Thyroid Section, Endocrine Division, Hospital de Clínicas de Porto Alegre, Federal University of Rio Grande do Sul, Porto Alegre, Brazil.

[3] Cardiovascular Aging Research Laboratory, Department of Kinesiology & Health Education, The University of Texas at Austin, Austin, TX USA.

* ORCID: https://orcid.org/0000-0002-5912-8020

Corresponding Author:
Dr Alexandre Machado Lehnen
Instituto de Cardiologia do Rio Grande do Sul
Av. Princesa Isabel, 395 Santana, 90620-001 Porto Alegre – RS Brazil
Phone: +55(51)32303600, branch 3636/3757
Fax: +55(51)32303600 branch 3757
amlehnen@gmail.com

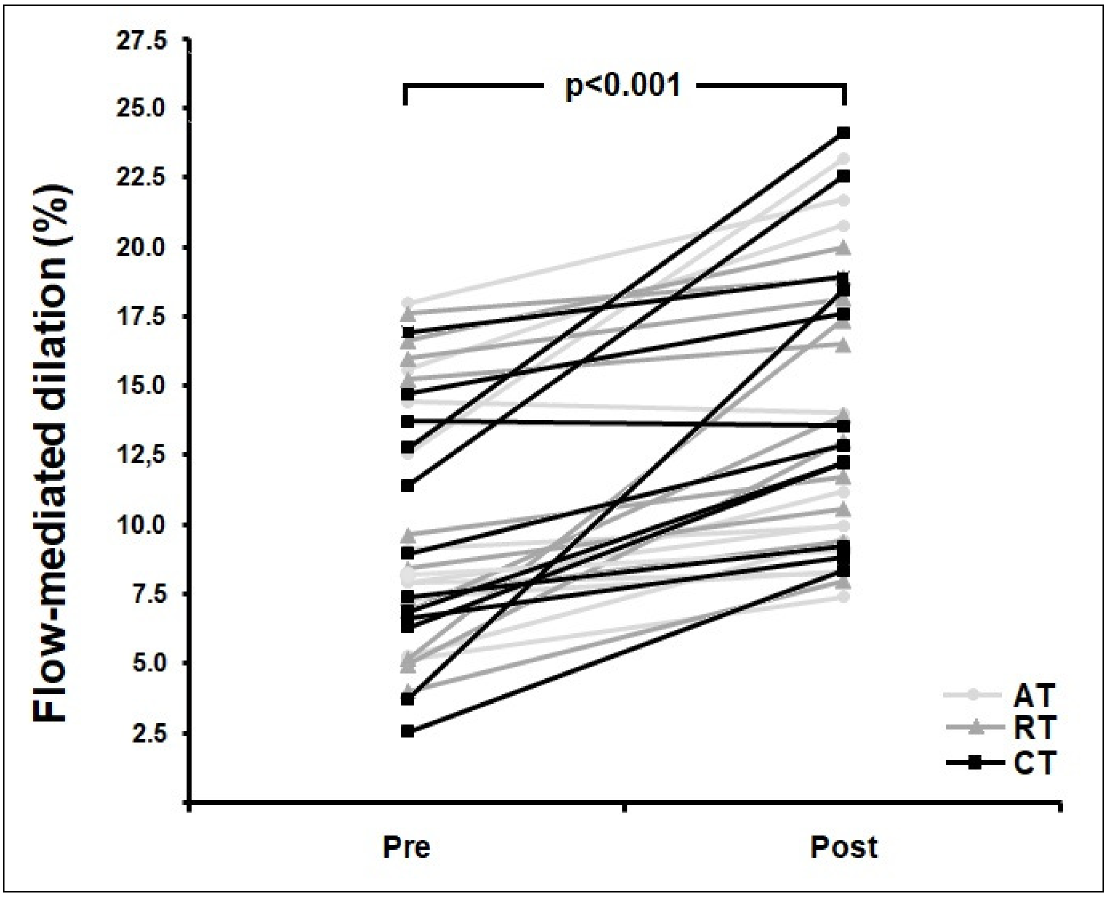

Figure S1 – Individual data points for Flow-mediated dilation measurements before (pre) and after (post) aerobic exercise training (AT), resistance training (RT), and combined training (CT).
